# The Germ Theory

**Published:** 1879-11

**Authors:** 


of disease seems to have become the sentiment of the scientific, philosophical and medical world, meaning simply that the cause of many diseases is the presence of living, organic existences in the blood, animal or vegetable, which have made their way into the circulation, through the medium of the stomach and lungs; but if a more etherial substance, destructive of this life, can be made to follow it into the circulation, whether through the stomach or the lungs, the theory and the practice are perfectly consistent.

Of a summer morning, there is more of ozone and less and less as the day advances. Our own senses tell us that this summer morning air is more full of life than that at noon. It is the greater amount of ozone in the mountain air, at the sea side and in the pine woods, which gives the greater pleasure and benefit in breathing.

In an ordinary atmosphere about one hundred thousandth part is Ozone; in the best mountain air there is about one ten thousandth part, and that difference in the amount of Ozone makes the difference in freshness and life of mountain air above the ordinary atmosphere.

"The Scientific American," in a recent editorial says of Ozone that "it is, 'par excellence,' that king among elements which subdues them to purposes of nature and life, exalted by electric force to a hight of aggressive energy which consumes decay and corruption, seems to attack the sensitive tissue in living organisms with a stimulating power that imparts through every organ the sense of refreshment and invigoration attendant upon the "clearing [Ozonizing] of the atmosphere" by a thunder storm. Its gradual disappearance from the atmosphere marks the approach of malignant epidemics, such as Asiatic cholera, and its appearance is the signal for their abatement. Dr. Moffat's observations of Ozone in the atmosphere before and during the cholera epidemics of 1853 at Newcastle and 1854 at London, established these coincidences with the greatest precision. The south wind that springs up at length, after a very stagnant and sickly season, and brings what we call purifying thunder showers, is proved to be an ozonized wind, and directly the starched paper in this wind feels the action of the liberated iodine and begins to change color, the epidemic begins to abate.

We have seen that common oxygen enters the circulation, much more will super oxygen, which is Ozone, do so. If the atmosphere is charged with a larger amount of Ozone, graduated by the scale mentioned, it must follow the insect life into the blood and utterly consume it, as also everything else in the blood which is foreign to it; in other words, it purifies the blood, and to that extent tends to cure all diseases which arise from impure blood; consequently removes all congestions, cures all congestive maladies. If then we shut a man up in a room, the atmosphere of which is purer than the mountain air, in consequence of its containing more Ozone, he must be greatly benefitted; and if again, water is impregnated with Ozone, it is clear that remedial means of great efficiency may be conveyed into the system through both the stomach and the lungs. Thoughtful German Physicians have taken these hints, and have applied them with favorable results; results sometimes most gratifying, not only for the promptness but the efficiency of the remedial action; relieving of severe pains within a few moments, surprisingly efficacious in gout, catarrh, chronic fever and ague, and the like. A German savan, a thorough medical scholar has, in a small and quiet way, been making a practical application of these remedial means in this city, to catarrh, nasal, bronchial and intestinal, as well as to other forms of disease; more in search of new truth than making money, but with this exception, no one else in the whole country is known to have acted on a suggestion which appears of such great practical value.

Any room can be charged with an ozonized atmosphere, even more life-giving than the purest air from the mountains; and while supplied with books, magazines, periodicals, paintings, and the means of engaging in a great variety of games and pastimes, the invalid is doing more for the restoration of his health, than if he went to the mountain tops, the sea shore, pine woods, or to the Sunny South and the islands of the Gulf, without the disadvantages, exposure and expense of leaving home for weeks and months at a time.

The argument is, that certain diseases are caused by living things, vegetable or animal, entering into the blood, called by various names, as Monads, Vibrios, Bacterides, Infusorii, Zoophites, Fungi and Algæ :

That air containing oxygen enters and mixes with the blood, carrying with it more or less of ozone, and which, coming in contact with these living things, oxydizes them, literally burning them up. If there is but little ozone present, but few of them are consumed and persons remain feeble, but if there is a great deal of ozone present, as in the air of the mountain, the sea and the pine woods, and this air is breathed for a considerable time, all those organ.sms are eventually burned up, the health improving in proportion.

If then, Ozone is the great and powerful agent which it is claimed to be, powerful in consuming whatever is in the blood which causes disease, and if it can be generated cheaply, and in any desired qaantity, an atmosphere can be impregnated with it to a greatly larger extent than is natural, this artificial ozonized air is proportionately more powerful to cure certain diseases and to restore health, and will do it in much less time than mountain or sea air.

Ozone must find its way into the blood, because oxygen, of which it is a modification, is known to do so. Schmidt, of Dorpat, has demonstrated the presence of Ozone in the blood, and both Schonbein and Hiss have shown that Ozone inhaled is absorbed by the blood, and is immediately brought into contact with oxydizable matter.

But the production of Ozone being very expensive, its application was necessarily very limited. Ozone has 1.5 times the density of common oxygen and consequently 3 atoms of original oxygen in 1 molecule, and its powerful property consists in its tendency to cede the third atom of oxygen directly to the oxydizable matter, leaving common oxygen behind.

| [ o o ] | [ [o o] o ] |
|---|---|
| Common Oxygen. | Ozone. |

